# Dynamics Analysis of a Wireless Rechargeable Sensor Network for Virus Mutation Spreading

**DOI:** 10.3390/e23050572

**Published:** 2021-05-06

**Authors:** Guiyun Liu, Zhimin Peng, Zhongwei Liang, Junqiang Li, Lefeng Cheng

**Affiliations:** School of Machanical and Electric Engineering, Guangzhou University, Guangzhou 510006, China; liugy@gzhu.edu.cn (G.L.); JunqiangLi2021@163.com (J.L.); chenglefeng@gzhu.edu.cn (L.C.)

**Keywords:** WRSNs, mutation virus, stable analysis, optimal control

## Abstract

Virus spreading problems in wireless rechargeable sensor networks (WSNs) are becoming a hot topic, and the problem has been studied and discussed in recent years. Many epidemic spreading models have been introduced for revealing how a virus spreads and how a virus is suppressed. However, most of them assumed the sensors are not rechargeable sensors. In addition, most of existing works do not consider virus mutation problems. This paper proposes a novel epidemic model, including susceptible, infected, variant, low-energy and dead states, which considers the rechargeable sensors and the virus mutation factor. The stability of the proposed model is first analyzed by adopting the characteristic equation and constructing Lyapunov functions methods. Then, an optimal control problem is formulated to control the virus spread and decrease the cost of the networks by applying Pontryagin’s maximum principle. Finally, all of the theoretical results are confirmed by numerical simulation.

## 1. Introduction

### 1.1. Research Background

In recent years, wireless sensor networks (WSNs) have played a crucial role in the internet of things (IoT), and WSNs has been widely developed and applied in many fields, for example: industrial, military and healthcare applications. Benefiting from the recent breakthrough in WSNs, they have attracted increasingly attention [1,2]. WSNs encompass numerous sensor nodes and nodes connect with each other with the radio signal, but it is difficult to create a complex security protective structure. Due to the vulnerability, the network is always destroyed by malware and this leads to information leakage and even network paralysis. Thus, security is an essential problem in WSNs to ensure accuracy and efficiency. Scholars have done a lot of research on the security of WSNs [3,4,5,6,7].

WSNs suffer from the issue of the vulnerability of the network and energy limitation. Wireless rechargeable sensor networks (WRSNs) [8] are considered wireless power transfer (WPT) technology which greatly improve WSNs. The security of WSNs has been pushed to a new level by optimizing the charging scheduling and analyzing the results of denial of charge attacks [9,10]. It is popular to apply epidemic dynamics analysis in WRSNs attacked by malwares. The classical mathematical models have been researched by scholars, including susceptible, infected (SI), susceptible, infected, susceptible (SIS), susceptible, infected, removed (SIR), susceptible, exposed, infected, removed (SEIR), etc. However, the malware can be changed when the malware attacks the network [11], and the same is true for a virus mutation. Once the virus mutation happens in WRSNs, it is devastating, and the system of the security strategy is destroyed. However, it has seldom been researched by scholars before.

Thus, this paper includes virus mutation to establish a new model, which is very effective to reduce the spreading of the virus, reduce the harm of the virus mutation to WRSNs and greatly improve the security of WRSNs.

### 1.2. Related Work

In order to improve the security in WRSNs, the efficient scheme and energy efficient secured ring routing (E2SR2) protocol was first proposed by Shafie et al. and Bhushan et al. [12,13]. For the problem of physical node capture attacks, a response strategy was proposed by Bonaci et al. [6] to ensure security and stability of the network connectivity when the network is attacked. Considering detection and correction, Sing et al. [3] also proposed a selective forwarding attacks technology which increases the QOS and provides better data transmission.

Applying epidemic dynamics analysis to the study of WSNs, Kephart et al. [14,15] firstly proposed a model to study and predict the spread of the virus. Since then, many new models have been proposed to study the problem. Cao et al. [16] considered the recovered factor to construct the model of WSNs, and the authors patched the dissemination of security and immunized or healed the nodes to use the security strategy; Zheng et al. [17] considered the vaccination strategies with temporary immunity and a quarantined strategy to construct an SEIQR model; Han et al. [18] considered the system of the nonlinear stochastic system to construct an SEIR model. Liu et al. [19] considered the low-energy factor to construct a model of WRSNs, and the stability of the model was proved. According to Liu et al. [19,20,21,22], the system is a good combination of an epidemic dynamic model and wireless sensor network. Thus, the system skillfully blends the information of the WRSNs with biological characteristics. However, the all of above models do not consider the problem of virus mutation. Above, Table 1 lists the recent relevant studies. 

As shown in Table 1, based on the virus mutation, scholars have done some studies. However, as we all know, the theories of the epidemic dynamics with virus mutation applied in WRSNs are rarely studied. The infected nodes infect other nodes to cause mutations because of coding errors, decryption problems, etc. [11], hence, the idea of virus mutation of artificial life was introduced, and a new way needs to be proposed to analyze and solve the security of the mutation virus model. Thus, in this paper, a new scheme is put forward of a model combined with virus mutation and is analyzed to solve the WRSN spreading problem of a mutated virus.

### 1.3. Contributions

In the previous studies, the charging behavior and mutation virus activity of wireless sensor networks has rarely been investigated. In this paper, the main goal is to introduce a model that considers the low-energy states and virus mutation. Combined with a practical application, the system can be applied when the stability of the system is proven and the other conditions are not taken into account, and the cost can be controlled by an optimal strategy. It is divided into five variable states. Our contributions are as follows:

1. An epidemic model suitable for WRSNs is established to describe the propagation process of malwares (the virus and mutated virus).
2. To analyze and calculate the basic reproductive numbers $R_{1}$ and $R_{2}$. Then, considering the existence of equilibrium of the system, the local and global asymptotic stability is proved by adopting the characteristic equation and the Lyapunov principle. Numerical simulation is carried out to confirm the results.
3. By constructing the objective function and applying Pontryagin’s maximum principle, we can obtain the optimal control variable which satisfies the optimal control objective of the security problem.

The rest of the paper is as follows: In Section 2, the introduction and analysis of the model is presented; in Section 3, the local and global asymptotic stability is proved; in Section 4, the optimal strategy is presented; in Section 5, the numerical simulation verifies the proposed theoretical results; in Section 6, the relevant conclusion of the model is presented.

## 2. Epidemic Modeling

### 2.1. Model Analysis

**Figure 1:**
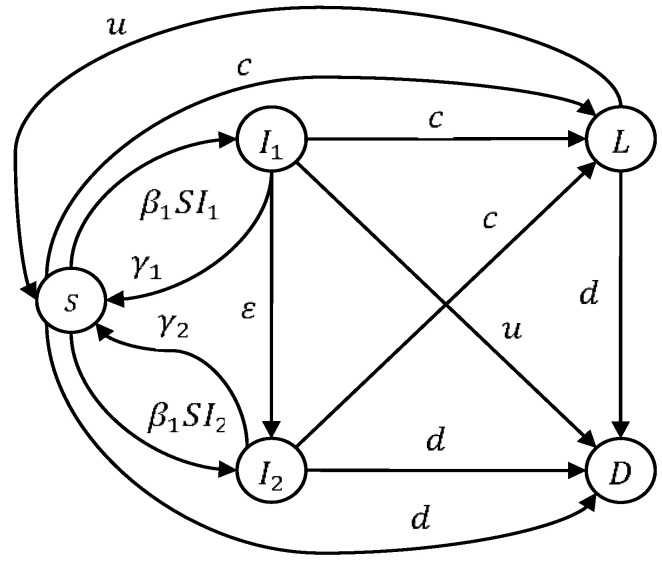
State transition diagram of the model.

In this section, the epidemic model of virus mutation in WRSNs is introduced. Sensors are divided into five sensor node states. The five sensor node states include: S, $I_{1}$, $I_{2}$, L and D. The S state is susceptible to virus attacks.$\;I_{1}$ is the infected node state,$\;I_{2}$ is the mutant virus node state, L is the low-energy state of all nodes, D represents death nodes (the sensor nodes cannot work). N stands for the sum of all nodes, and it is a certain number. Thus, $N\left(t\right)=S\left(t\right)+I_{1}\left(t\right)+I_{2}\left(t\right)+L\left(t\right)$. The state transition diagram is shown in Figure 1.

As shown in Figure 1, A is the number of injected nodes; $\beta_{1}$ is the conversion rate of susceptible to infected; $\beta_{2}$ is the conversion rate of the sensor nodes becoming infected with the virus when the virus mutates; $\gamma_{1}$ is the rate of cleaning $I_{1}$ virus; $\gamma_{2}$ is the rate of cleaning $I_{2}$ virus; d is the conversion rate when sensors are unable to work; c is the rate of nodes going into a low-energy state; u is the rate of recovery from L energy, and during the low-energy state, $I_{1}$ and $I_{2}$ have removed the virus; ε is the rate of virus mutation. In this paper, when infected nodes and mutant virus nodes are converted to nodes of the low-energy state, a virus remover is created to eliminate the virus and mutant virus. According to the parameters and nodes of states, the dynamics equation of the model (1) is given as the following system (1):

(1) $$\{\begin{array}{c} \frac{dS\left(t\right)}{dt}=A-\left(d+c\right)S\left(t\right)-\left(\beta_{1}S\left(t\right)-\gamma_{1}\right)I_{1}\left(t\right)-\left(\beta_{2}S\left(t\right)+\gamma_{2}\right)I_{2}\left(t\right)+uL\left(t\right)\\ \frac{dI_{1}\left(t\right)}{dt}=\beta_{1}S\left(t\right)I_{1}\left(t\right)-\gamma_{1}I_{1}\left(t\right)-cI_{1}\left(t\right)-\varepsilon I_{1}\left(t\right)-dI_{1}\;\left(t\right)\;\;\\ \frac{dI_{2}\left(t\right)}{dt}=\beta_{2}S\left(t\right)I_{2}\left(t\right)-\gamma_{2}I_{2}\left(t\right)-cI_{2}\left(t\right)-dI_{2}\left(t\right)+\varepsilon I_{1}\left(t\right)\;\;\;\\ \frac{dL\left(t\right)}{dt}=c\left(I_{1}\left(t\right)+I_{2}\left(t\right)+S\left(t\right)\right)-uL\left(t\right)-dL\left(t\right) \end{array}$$

Obviously, adding Equation (1), we obtain:

(2) $$\frac{dS\left(t\right)}{dt}+\frac{dI_{1}\left(t\right)}{dt}+\frac{dI_{2}\left(t\right)}{dt}+\frac{dL\left(t\right)}{dt}=A-d\left(S\left(t\right)+I_{1}\;\left(t\right)+I_{2}\left(t\right)+L\left(t\right)\right)$$

As $N\left(t\right)=S\left(t\right)+I_{1}\left(t\right)+I_{2}\left(t\right)+L\left(t\right)$, Equation (2) is given by:

(3) $$\frac{dN\left(t\right)}{dt}=A-dN\left(t\right)$$

Then, considering the limit of (3), we can obtain $N\left(t\right)\leq \frac{A}{d}$, so the feasible region for (1) is given by:

(4) $$\Psi =\left\{\left(S,I_{1},I_{2},L\right)\in {\mathbb{R}}^{4}|\;S\left(t\right)+I_{1}\;\left(t\right)+I_{2}\left(t\right)+L\left(t\right)\leq \frac{A}{d}\right\}$$

Above, Ψ is a positively invariant.

### 2.2. Computing the Equilibrium Points and Basic Reproductive Number

In order to discuss the existence of equilibria, let the left-hand side of Equation (1) be zero. Obviously, there are three solutions that satisfy this situation. The disease-free equilibrium is ($S_{0},0,0,L_{0}),$ the individual plant virus equilibrium is ($\bar{S},0,{\bar{I}}_{2},\bar{L}$) and the endemic equilibrium is ($S^{*},I_{1}^{*},I_{2}^{*},L^{*}).$ It is noted that the basic reproductive number determines the existence of different equilibria. In this paper, there are two basic reproductive numbers ($R_{1}$ and $R_{2}$), where $R_{1}=\frac{S_{0}}{S^{*}}$, $R_{2}=\frac{S_{0}}{\bar{S}}$. $S_{0}$, $\bar{S}$ and $S^{*}$ are given as:

(5) $$S_{0}=\frac{A}{d+c-\frac{uc}{u+d}}$$

(6) $$\bar{S}=\frac{\gamma_{2}+c+d}{\beta_{2}}$$

(7) $$S^{*}=\frac{\gamma_{1}+c+\varepsilon +d}{\beta_{1}}\;\;$$

Thus, the basics reproductive numbers are given as:

(8) $$\;R_{1}=\frac{\beta_{1}S_{0}}{\gamma_{1}+c+\varepsilon +d}\;R_{2}=\frac{\beta_{1}S_{0}}{\gamma_{2}+c+d}$$

According to the existence of the equilibrium point, if $R_{1}\leq 1$, $R_{2}\leq 1$, the system (1) has only a disease-free equilibrium point $E_{0}=\left(S_{0},0,0,L_{0}\right)$ given as:

(9) $$L_{0}=\frac{cS_{0}}{u+d}=\frac{Ac}{c+d-uc}$$

If $R_{2}>1$, the system has a disease-free point and an individual plant virus equilibrium point, and the individual plant virus equilibrium $\bar{E}=$($\bar{S},0,{\bar{I}}_{2},\bar{L}$) is given as:

(10) $$\bar{L}=\frac{c\left({\bar{I}}_{2}+\bar{S}\right)}{u+d}\;\;\;$$

(11) $${\bar{I}}_{2}=\frac{A-\left(d+c\right)\bar{S}+u\bar{L}}{\beta_{2}\bar{S}-\gamma_{2}}=\frac{S_{0}\left(R_{2}-1\right)}{R_{2}}$$

If $R_{1}>1$, $R_{1}>R_{2}$, the system has a disease-free equilibrium point and an endemic equilibrium point. The endemic equilibrium $E^{*}=\left(S^{*},I_{1}^{*},I_{2}^{*},L^{*}\right)$ is given as:

(12) $$I_{1}^{*}=\frac{S_{0}\beta_{2}\left(R_{1}-1\right)\left(R_{1}-R_{2}\right)}{R_{1}\left(\beta_{2}\left(R_{1}-R_{2}\right)+\frac{\varepsilon R_{1}R_{2}}{S_{0}}\right)}$$

(13) $$I_{2}^{*}=\frac{\varepsilon R_{1}R_{2}I_{1}^{*}}{S_{0}\beta_{2}\left(R_{1}-1\right)}$$

(14) $$L^{*}=\frac{c\left(I_{1}^{*}+I_{2}^{*}+S^{*}\right)}{u+d}$$

Thus, we have the following Theorem 1:

**Theorem** **1.** *If*$R_{1}\leq 1$*,*$R_{2}\leq 1$*, there is only one equilibrium*$E_{0}$*; if*$R_{2}>1$*, there are two equilibriums including*$E_{0}$*and*$\bar{E}$*; if*$R_{1}>1$*,*$R_{1}>R_{2}$*, there are two equilibriums including*$E_{0}$*and*$E^{*}$.

## 3. Dynamic Stability Analysis

In this section, the epidemic model of virus mutation in WRSNs is divided into local and global stability analysis.

System (1) is a nonlinear system, and we must turn it into a linear system so as to prove the local stability of this system. In order to reduce the calculated amount of the system, L in the first equation of the system (1) can be replaced with$\;N\left(t\right)-\left(S\left(t\right)+I_{1}\left(t\right)+I_{2}\left(t\right)\right)$. Thus, in system (1), the last equation is independent of the other 3 equations, and the linear equations can be given as the system (15):

(15) $$\{\begin{array}{c} \frac{dS}{dt}=J_{11}\left(S-S^{+}\right)+J_{12}\left(I_{1}-I_{1}^{+}\right)+J_{13}\left(I_{2}-I_{2}^{+}\right)\;\\ \frac{dI_{1}}{dt}=J_{21}\left(S-S^{+}\right)+J_{22}\left(I_{1}-I_{1}^{+}\right)+J_{23}\left(I_{2}-I_{2}^{+}\right)\;\\ \frac{dI_{2}}{dt}=J_{31}\left(S-S^{+}\right)+J_{32}\left(I_{1}-I_{1}^{+}\right)+J_{33}\left(I_{2}-I_{2}^{+}\right)\; \end{array}$$

Then, the Jacobian matrix of (15) is given by:

(16) $$J\left(E^{+}\right)=\left(\begin{array}{ccc} -\left(d+c+u\right)-\beta_{1}I_{1}-\beta_{2}I_{2} & -\beta_{1}S+\gamma_{1}-u & -\beta_{2}S+\gamma_{2}-u\\ \beta_{1}I_{1} & \beta_{1}S-\left(\gamma_{1}+c+\varepsilon +d\right) & 0\\ \beta_{2}I_{2} & \varepsilon & \beta_{2}S-\left(\gamma_{2}+c+d\right) \end{array}\right)$$

### 3.1. Local Stability

**Theorem** **2.** *If* $R_{1}\leq 1$ *and* $R_{2}\leq 1$ *, the disease-free equilibrium is locally asymptotically stable.*

**Proof.**  According to the Lyapunov criterion [31], if all of the characteristic values are negative, the equilibrium is locally asymptotically stable. Thus, the Jacobian matrix is given by:

(17) $$J\left(E_{0}\right)=\left(\begin{array}{ccc} -\left(d+c+u\right) & -\beta_{1}S_{0}+\gamma_{1}-u & -\beta_{2}S_{0}+\gamma_{2}-u\\ 0 & \beta_{1}S_{0}-\left(\gamma_{1}+c+\varepsilon +d\right) & 0\\ 0 & \varepsilon & \beta_{2}S_{0}-\left(\gamma_{2}+c+d\right) \end{array}\right)$$

Hence, we have the following characteristic Equation (18):

(18) $$f_{1}\left(\lambda \right)=\left(\lambda +\left(d+c+u\right)\right)\left(\lambda -\beta_{1}S_{0}+\gamma_{1}+c+\varepsilon +d\right)\left(\lambda -\beta_{2}S_{0}+\gamma_{2}+c+d\right)\;\;$$

Obviously, if $R_{1}\leq 1$ and $R_{2}\leq 1$, we have $\lambda_{1}=-\left(d+c+u\right)$,$\lambda_{2}=\beta_{1}S_{0}-\left(\gamma_{1}+c+\varepsilon +d\right)$ and $\lambda_{3}=\beta_{2}S_{0}-\left(\gamma_{2}+c+d\right)$. All of the characteristic values are negative, so the disease-free equilibrium is locally asymptotically stable. □

**Theorem** **3.** *If* $R_{2}>1$ *and* $R_{2}>R_{1}$ *, the individual plant virus equilibrium is locally asymptotically stable.*

**Proof.**  The Jacobian matrix is given as:

(19) $$J\left(\bar{E}\right)=\left(\begin{array}{ccc} -\left(d+c+u\right)-\beta_{2}{\bar{I}}_{2} & -\beta_{1}\bar{S}+\gamma_{1}-u & -\beta_{2}\bar{S}+\gamma_{2}-u\\ 0 & \beta_{1}\bar{S}-\left(\gamma_{1}+c+\varepsilon +d\right) & 0\\ \beta_{2}{\bar{S}}_{2} & \varepsilon & \beta_{2}\bar{S}-\left(\gamma_{2}+c+d\right) \end{array}\right)$$

Hence, the characteristic equation is given by:

(20) $$f_{2}\left(\lambda \right)=\left(\lambda ++d+c+u\right)\left(\lambda -\beta_{1}\bar{S}+\gamma_{1}+c+\varepsilon +d\right)\left(\lambda +\beta_{1}{\bar{I}}_{1}\right)$$

Then, the characteristic value is $\lambda_{1}=-\left(d+c+u\right)$, $\lambda_{2}$=$\beta_{1}\bar{S}-\left(\gamma_{1}+c+\varepsilon +d\right)$, $\lambda_{3}=-\beta_{2}{\bar{I}}_{2}$. Obviously, if $R_{2}>R_{1}$, $\lambda_{2}$ is always a negative number. Hence, the individual plant virus equilibrium is locally asymptotically stable. □

**Theorem** **4.** *If* $R_{1}>1$ *and* $R_{1}>R_{2}$ *, the endemic equilibrium is locally asymptotically stable.*

**Proof.**  The Jacobian matrix is given by:

(21) $$J\left(E^{*}\right)=\left(\begin{array}{ccc} -\left(d+c+u\right)-\beta_{1}I_{1}^{*}-\beta_{2}I_{2}^{*} & -\beta_{1}S^{*}+\gamma_{1}-u & -\beta_{2}S^{*}+\gamma_{2}-u\\ \beta_{1}I_{1}^{*} & \beta_{1}S^{*}-\left(\gamma_{1}+c+\varepsilon +d\right) & 0\\ \beta_{2}I_{2}^{*} & \varepsilon & \beta_{2}S^{*}-\left(\gamma_{2}+c+d\right) \end{array}\right)\;\;$$

By the transformation of (21), we obtain

(22) $$J\left(E^{*}\right)=\left(\begin{array}{ccc} -\left(d+c+u\right) & -\left(d+c+u\right) & -\left(d+c+u\right)\\ \beta_{1}I_{1}^{*} & \beta_{1}S^{*}-\left(\gamma_{1}+c+\varepsilon +d\right) & 0\\ \beta_{2}I_{2}^{*} & \varepsilon & \beta_{2}S^{*}-\left(\gamma_{2}+c+d\right) \end{array}\right)$$

We have the following characteristic equation:

(23) $$f_{3}\left(\lambda \right)=\left(\lambda +d+c+u\right)(\lambda^{2}-\left(\left(\beta_{2}S^{*}-\left(\gamma_{2}+c+d\right)-\beta_{1}I_{1}^{*}\right)\lambda +\beta_{1}I_{1}^{*}\left(\gamma_{2}+d+c+\varepsilon -\beta_{2}S^{*}\right)\right)$$

Obviously, $\lambda_{1}=-\left(d+c+u\right)$, and the other characteristic values can be calculated by the following Equation (24):

(24) $$X\left(\lambda \right)=\lambda^{2}-\left(\left(\beta_{2}S^{*}-\left(\gamma_{2}+c+d\right)-\beta_{1}I_{1}^{*}\right)\lambda +\beta_{1}I_{1}^{*}\left(\gamma_{2}+d+c+\varepsilon -\beta_{2}S^{*}\right)\right)$$

According to Vieta’s formulas [32], the zero solution of (24) is given by $\lambda_{2}+\lambda_{3}=\beta_{2}S^{*}-\left(\gamma_{2}+c+d\right)-\beta_{1}I_{1}^{*}$, $\lambda_{2}*\lambda_{3}=\beta_{1}I_{1}^{*}*\left(\gamma_{2}+c+d+\varepsilon -\beta_{2}S^{*}\right)$, thus, if $R_{1}>1$ and $R_{1}>R_{2}$, the value of $\lambda_{2}$ and $\lambda_{3}$ can be proved to be $\lambda_{2}+\lambda_{3}<0$ and $\lambda_{2}*\lambda_{3}>0$. Hence, the endemic equilibrium is locally asymptotically stable. □

### 3.2. Global Stability

In this part, three Lyapunov functions are constructed to verify the global stability of the disease-free equilibrium $E_{0}$, individual plant virus equilibrium $\bar{E}$ and endemic equilibrium $E^{*}$.

**Theorem** **5.** *If* $R_{1}\leq 1$ *,* $R_{2}\leq 1$ *and* $R_{1}+\frac{\varepsilon }{\gamma_{1}+c+\varepsilon +d}<1$ *, the disease-free equilibrium is globally stable.*

**Proof.**  The Lyapunov function is constructed as $V\left(I_{1},I_{2}\right)$=$I_{1}+I_{2}$. We obtain

(25) $$\begin{array}{cc} \frac{dV}{dt} & =\frac{dI_{1}}{dt}+\frac{dI_{2}}{dt}\\ & =\left(\beta_{1}S-\left(\gamma_{1}+c+\varepsilon +d\right)\right)I_{1}+(\beta_{2}S-(\gamma_{2}+c+d))I_{2}+\varepsilon I_{1}\\ & \leq \left(\beta_{1}S_{0}-\left(\gamma_{1}+c+\varepsilon +d\right)\right)I_{1}+(\beta_{2}S_{0}-(\gamma_{2}+c+d))I_{2}+\varepsilon I_{1}\\ & =\left(\gamma_{1}+c+\varepsilon +d\right)\left(R_{1}-1+\frac{\varepsilon }{\gamma_{1}+c+\varepsilon +d}\right)I_{1}+(\gamma_{2}+c+d)\left(R_{2}-1\right)I_{2}\; \end{array}$$

Obviously, if $R_{1}+\frac{\varepsilon }{\gamma_{1}+c+\varepsilon +d}<1$ and$\;R_{2}<1$, the disease-free equilibrium is globally stable. □

**Theorem** **6.** *If* $R_{2}>1,R_{1}<1$ *, the individual plant virus equilibrium is globally stable.*

**Proof.**  According to the second Equation (1), the number of infected nodes is $I_{0}$ at $t_{0}(t>t_{0})$. We can obtain.

(26) $$I_{1}\left(t\right)=I_{0}e^{\left(\beta_{1}S-\left(\gamma_{1}+c+\varepsilon +d\right)\right)\left(t-t_{0}\right)}$$

When t→∞, $I_{1}\left(t\right)$ will be stable at zero. Thus, the limit Equation (27) is given as:

(27) $$\{\begin{array}{c} \frac{dS\left(t\right)}{dt}=A-\left(d+c+u\right)S\left(t\right)-\left(\beta_{2}S\left(t\right)+\gamma_{2}+u\right)I_{2}\left(t\right)+uL\left(t\right)\\ \frac{dI_{2}\left(t\right)}{dt}=\beta_{2}S\left(t\right)I_{2}\left(t\right)-\gamma_{2}I_{2}\left(t\right)-cI_{2}\left(t\right)-dI_{2}\left(t\right) \end{array}$$

As $I_{1}\left(t\right)$ converges to a limit value zero, the Lyapunov function can be given as:

(28) $$V\left(S,I_{2}\right)=\frac{1}{2}{\left(S-\bar{S}\right)}^{2}+\frac{\beta_{2}\bar{S}-\gamma_{2}+u}{\beta_{2}}\left(I_{2}-{\bar{I}}_{2}-{\bar{I}}_{2}ln\frac{I_{2}}{{\bar{I}}_{2}}\right)$$

The derivative of $V\left(S,I_{2}\right)$ of (28) is given by:

(29) $$\begin{array}{cc} \frac{dV}{dt} & =\left(S-\bar{S}\right)\frac{dS}{dt}+\frac{\beta_{2}\bar{S}-\gamma_{2}+u}{\beta_{2}}\frac{I_{2}-{\bar{I}}_{2}}{I_{2}}\frac{dI_{2}}{dt}\\ & =-(d+u+c+\beta_{2}I_{2}){\left(S-\bar{S}\right)}^{2}-\left(\beta_{2}\bar{S}-\gamma_{2}+u\right)\left(I_{2}-{\bar{I}}_{2}\right)\left(S-\bar{S}\right)\\ & +\frac{\beta_{2}\bar{S}-\gamma_{2}+u}{\beta_{2}}(\beta_{2}\left(\left(I_{2}-{\bar{I}}_{2}\right)\left(S-\bar{S}\right)\right)\\ & =-\left(d+u+c+\beta_{2}I_{2}\right){\left(S-\bar{S}\right)}^{2} \end{array}$$

Obviously, if$\;R_{2}>1,R_{1}<1$, the individual plant virus equilibrium is globally stable. □

**Theorem** **7.** *If* $R_{1}>1,R_{1}>R_{2}$ *and* ε=0 *, the endemic equilibrium is globally stable.*

**Proof.**  It is noted that the rate of infection is higher than the rate of virus mutation. If the rate of virus mutation is enough small and is equal to be zero, it is assumed that ε=0. The Equation (1) is given as:

(30) $$\begin{array}{c} \frac{dS\left(t\right)}{dt}=A-\left(d+c\right)S\left(t\right)-\left(\beta_{1}S\left(t\right)-\gamma_{1}\right)I_{1}\left(t\right)-\left(\beta_{2}S\left(t\right)+\gamma_{2}\right)I_{2}\left(t\right)+uL\left(t\right)\\ \frac{dI_{1}\left(t\right)}{dt}=\beta_{1}S\left(t\right)I_{1}\left(t\right)-\gamma_{1}I_{1}\left(t\right)-cI_{1}\left(t\right)-dI_{1}\;\left(t\right)\\ \frac{dI_{2}\left(t\right)}{dt}=\beta_{2}S\left(t\right)I_{2}\left(t\right)-\gamma_{2}I_{2}\left(t\right)-cI_{2}\left(t\right)-dI_{2}\left(t\right)\;\\ \frac{dL\left(t\right)}{dt}=c\left(I_{1}\left(t\right)+I_{2}\left(t\right)+S\left(t\right)\right)-uL\left(t\right)-dL\left(t\right) \end{array}$$

The Lyapunov function $V\left(S,I_{1},I_{2}\right)$ is given as follows:

(31) $$V\left(S,I_{1},I_{2}\right)=\frac{1}{2}{\left(S-S^{*}\right)}^{2}+\frac{c+d}{\beta_{1}}\left(I_{1}-I_{1}^{*}-I_{1}^{*}ln\frac{I_{1}}{I_{1}^{*}}\right)+\frac{c+d}{\beta_{2}}\left(I_{2}-I_{2}^{*}-I_{2}^{*}ln\frac{I_{2}}{I_{2}^{*}}\right)$$

Obviously, $V\left(S,I_{1},I_{2}\right)\geq 0$. Then, the derivative of $V\left(S,I_{1},I_{2}\right)$ of the solution of (31) is given by:

$$\frac{dV}{dt}=-\left(d+c+u+\beta_{1}I_{1}+\beta_{2}I_{2}\right){\left(S-S^{*}\right)}^{2}+\left(\beta_{1}S^{*}-\left(\gamma_{1}+c+d\right)\right){\left(I_{1}-I_{1}^{*}\right)}^{2}+\left(\beta_{2}S^{*}-\left(\gamma_{1}+c+d\right)\right){\left(I_{2}-I_{2}^{*}\right)}^{2}\;=-\left(d+c+u+\beta_{1}I_{1}+\beta_{2}I_{2}\right){\left(S-S^{*}\right)}^{2}+\left(\beta_{2}S^{*}-\left(\gamma_{1}+c+d\right)\right){\left(I_{2}-I_{2}^{*}\right)}^{2}\;$$

If $R_{1}>R_{2}$, the endemic equilibrium is globally stable if ε=0. Theorem 6 is proved. □

## 4. Optimal Strategy

In this subsection, the cost of cleaning the virus and the cost of charging low-energy nodes are considered. For this purpose, the control U(·) is introduced as the objective function, $\gamma_{1}(0<\gamma_{1}<1)$ is the rate of cleaning $I_{1}$ virus; $\gamma_{2}(0<\gamma_{2}<1)$ is the rate of cleaning $I_{2}$ virus and u(0<u<1) is the rate of recovery energy. $Q_{I_{1}}$ is the treatment cost coefficient of $I_{1}$, $Q_{I_{2}}$ is the treatment cost coefficient of $I_{2}$ and $Q_{L}$ is the charging cost coefficient of low-energy nodes. Thus, the optimal control problem is to minimize the objective function as follows:

(32) $$U\left(\gamma_{1},\gamma_{2},u\right)=min\left\{I_{1}\left(tf\right)+I_{2}\left(tf\right)+L\left(tf\right)+\overset{tf}{\underset{0}{\int }}(Q_{I_{1}}{\left(\gamma_{1}I_{1}\right)}^{2}+Q_{I_{2}}{\left(\gamma_{2}I_{2}\right)}^{2}+Q_{L}\left(uL{)}^{2}\right)dt\right\}$$

It is clear that the feasible region of the control variable set U of the system is [0, 1]. Hence, the optimization goal is to diminish $I_{1}$ and $I_{2}$ during the time interval [0,tf]. According to the Pontryagin maximum principle, the Hamiltonian function is constructed as follows:

(33) $$H\left(X,U,\alpha ,t\right)=Q_{I_{1}}\gamma_{1}^{2}I_{1}^{2}+Q_{I_{2}}\gamma_{2}^{2}I_{2}^{2}+Q_{L}u^{2}L^{2}+\alpha_{1}\frac{dS}{dt}+\alpha_{2}\frac{dI_{1}}{dt}+\alpha_{3}\frac{dI_{2}}{dt}+\alpha_{4}\frac{dL}{dt}$$

X is the state variable, U is the control variable set, $\alpha_{i}\left(i=1,,2,3,4\right)$ is the adjoint variables. The adjoint variables are determined by solving the following equations.

(34) $$\left\{ \begin{array}{c} \frac{d\alpha_{1}}{dt}=-\frac{\partial H}{\partial S}=\alpha_{1}\left(t\right)\left(d+c+\beta_{1}I_{1}+\beta_{2}I_{2}\right)-(\alpha_{2}\left(t\right)\beta_{1}I_{1}+\alpha_{3}\left(t\right)\beta_{2}I_{2}+\alpha_{4}\left(t\right)c)\;\\ \frac{d\alpha_{2}}{dt}=-\frac{\partial H}{\partial I_{1}}=-2Q_{I_{1}}\gamma_{1}^{2}I_{1}-(\alpha_{1}\left(t\right)\left(\gamma_{1}-\beta_{1}S\right)+\alpha_{2}\left(t\right)\left(\beta_{1}S-\left(\gamma_{1}+c+\varepsilon +d\right)\right)+\alpha_{3}\left(t\right)\varepsilon +\alpha_{4}\left(t\right)\\ \frac{d\alpha_{3}}{dt}=-2Q_{I_{2}}\gamma_{2}^{2}I_{2}-(\alpha_{1}\left(t\right)\left(\gamma_{2}-\beta_{2}S\right)+\alpha_{3}\left(t\right)\left(\beta_{2}S-\left(\gamma_{2}+c+d\right)\right)+\alpha_{4}\left(t\right)c\;\\ \frac{d\alpha_{4}}{dt}=-\frac{\partial H}{\partial L}=-2Q_{L}u^{2}L-\left(\alpha_{1}\left(t\right)u-\alpha_{4}\left(t\right)\left(u+d\right)\right) \end{array}\right.$$

At the moment, the transversality condition of optimal control satisfies the following equation:

(35) $$\alpha_{1}\left(tf\right)=0;\;\alpha_{2}\left(tf\right)=1;\;\alpha_{3}\left(tf\right)=1;\;\alpha_{4}\left(t\right)=1$$

According to differential equations of the covariant variable and transversal condition of optimal control, the optimization condition is given by:

(36) $$\frac{\partial H}{\partial \gamma_{1}}=2Q_{I_{1}}\gamma_{1}I_{1}^{2}+\alpha_{1}\left(t\right)I_{1}-\alpha_{2}\left(t\right)I_{1}\;\frac{\partial H}{\partial \gamma_{2}}=2Q_{I_{2}}\gamma_{2}I_{2}^{2}+\alpha_{1}\left(t\right)I_{2}-\alpha_{3}\left(t\right)I_{2}\;\frac{\partial H}{\partial u}=2Q_{L}uL^{2}+\alpha_{1}\left(t\right)L-\alpha_{4}\left(t\right)L\;\;$$

Finally, Pontryagin’s maximum principle is applied to obtain the optimal control variable set. The result is given as:

(37) $$\left\{ \begin{array}{c} \gamma_{1}=min\left\{\left(\max\left(0,\frac{\alpha_{2}\left(t\right)-\alpha_{1}\left(t\right)}{2Q_{I_{1}}\gamma_{1}I_{1}}\right),1\right)\right\}\\ \gamma_{2}=min\left\{\left(\max\left(0,\frac{\alpha_{3}\left(t\right)-\alpha_{1}\left(t\right)}{2Q_{I_{2}}\gamma_{2}I_{2}}\right),1\right)\right\}\\ u=min\left\{\left(\max\left(0,\frac{\alpha_{4}\left(t\right)-\alpha_{1}\left(t\right)}{2Q_{L}uL}\right),1\right)\right\} \end{array}\right.$$

## 5. Numerical Simulation

In this section, all of the theoretical analyses are proved and the numerical results of the system (1) are presented to support the analytic results. The result of the numerical simulation is given as follows.

### 5.1. Stability Simulation

**Figure 2:**
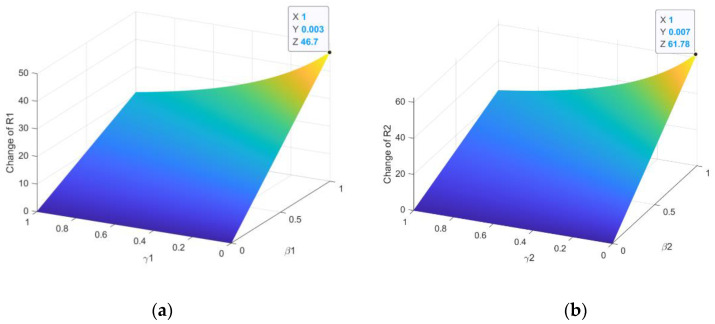
Relation of parameters and the basic reproduction number: (**a**) Relationship between $R_{1}$ and $\beta_{1}$, $\gamma_{1}$; (**b**) relationship between $R_{2}$ and $\beta_{2}$, $\gamma_{2}$

The parameters are given so that A=10, d=0.1, c=0.5, u=0.2, ε=0.2. It can be seen that rates of $\beta_{1}$ and $\gamma_{1}$ have significant impacts on the basic reproduction number $R_{1}$, shown in Figure 2a. With the increasing of $\beta_{1}$, $R_{1}$ increases rapidly, and with the increasing of $\gamma_{1}$, $R_{1}$ increases slowly. In the same way, the rates of $\beta_{2}$ and $\gamma_{2}$ also have significant impacts on the basic reproduction number $R_{2}$, shown in Figure 2b. With the increasing of $\beta_{2}$, $R_{2}$ increases rapidly, and with the increasing of $\gamma_{2}$, $R_{2}$ increases slowly.

**Figure 3:**
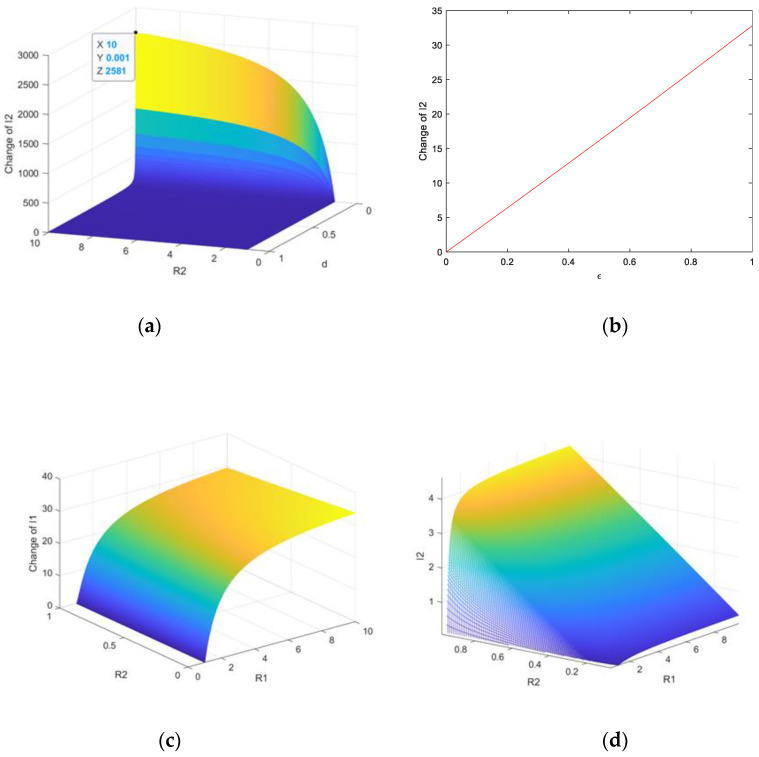
Relationship between $I_{1}$, $I_{2}$, the basic reproductive number: (**a**) Relationship between ${\bar{I}}_{2}$ and $d,\;R_{2}$; (**b**) relationship between $I_{2}^{*}$ and ε; (**c**) relationship between $I_{1}$ and $R_{1}$, $R_{2}$; (**d**) relationship between $I_{2}$ and $R_{1}$, $R_{2}$

The relationship between $R_{1}$, $R_{2}$ and equilibrium is shown in Figure 3. The parameters of the system (1) are set as A=10, c=0.5, u=0.2. It is noted that the individual plant virus equilibrium ${\bar{I}}_{2}$ is determined by d and the basic reproduction number $R_{2}$. The rate of death d has significant impacts on the ${\bar{I}}_{2}$ if d is small enough, as shown in Figure 3a. It can be seen that the relationship between the rate of virus mutation ε and $I_{2}^{*}$ is a linear, as shown in Figure 3b. The relationships among $R_{1}$, $R_{2}$ and $I_{1}$, $I_{2}$ are shown in Figure 3c,d when the system (1) satisfies the conditions of the existence of endemic equilibrium $\left(R_{1}>1,R_{1}>R_{2}\right)$.

**Figure 4:**
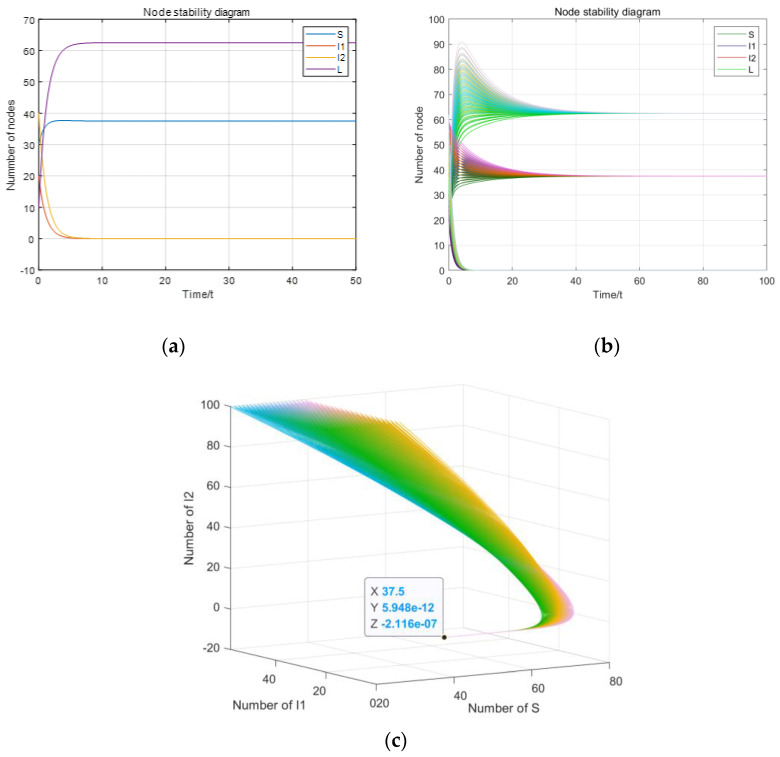
Diagram of the disease-free equilibrium: (**a**) Stable value of disease-free equilibrium; (**b**) the stability of changes to the initial value; (**c**) three-dimensional diagram of stability of changes to the initial value.

If R_1_ < 1 and R_2_ < 1, the parameters are set as β_1_ = 0.003, β_2_ = 0.002, γ_1_ = 0.2, γ_2_ = 0.4, c = 0.5, u = 0.2, d = 0.1, ε = 0.2 and A = 10. Hence, the basic reproductive numbers are calculated as R_1_ = 0.1125 < 1 and R_2_ = 0.075 < 1. N = S + I_1_ + I_2_ + L = 100. The disease-free equilibrium E_0_ (S_0_, 0, 0, L_0_) can been calculated as S_0_ = 37.5, L_0_ = 62.5, I_1_ = 0, I_2_ = 0, as shown in Figure 4.

As shown in Figure 4a–c, according to Theorem 2, the initial values S(0), I_1_(0), I_2_(0) and L(0) do not have any influence on system stability and the system (1) will be stable at the disease-free equilibrium E_0_ eventually.

**Figure 5:**
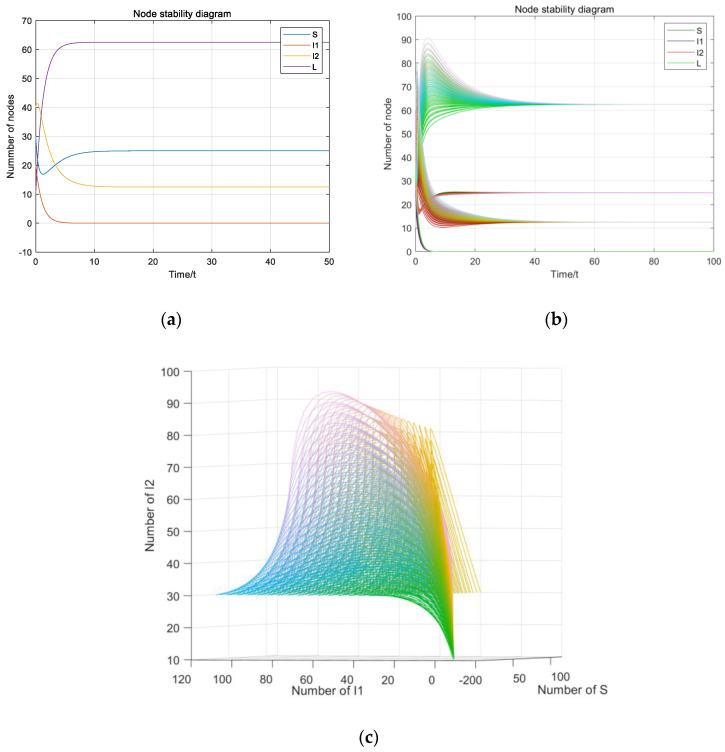
The individual plant virus equilibrium: (**a**) Stable value of individual plant virus equilibrium; (**b**) the stability of changes to the initial value; (**c**) three-dimensional diagram of stability of changes to the initial value.

If the parameters satisfy R_2_ > 1 and R_2_ > R_1_, β_1_ = 0.003, β_2_ = 0.04, γ_1_ = 0.2, γ_2_ = 0.4, c = 0.5, u = 0.2, d = 0.1, ε = 0.2, A = 10, we have R_1_ = 0.1125 < 1 and R_2_ = 1.5 > 1 > R_1_. The individual plant virus equilibrium is calculated to be $\bar{E}(\bar{S},0,{\bar{I}}_{2},\bar{L})$, $\bar{S}=25,\;{\bar{I}}_{2}=12.5,\;\bar{L}=62.5$, as shown in Figure 5.

According to Theorem 3, it is noted that the system (1) will be stable at the individual plant virus equilibrium eventually. From Figure 5a–c, the initial values do not have any influence on system stability and the system (1) will be stable at the individual plant virus equilibrium $\bar{E}$ eventually.

**Figure 6:**
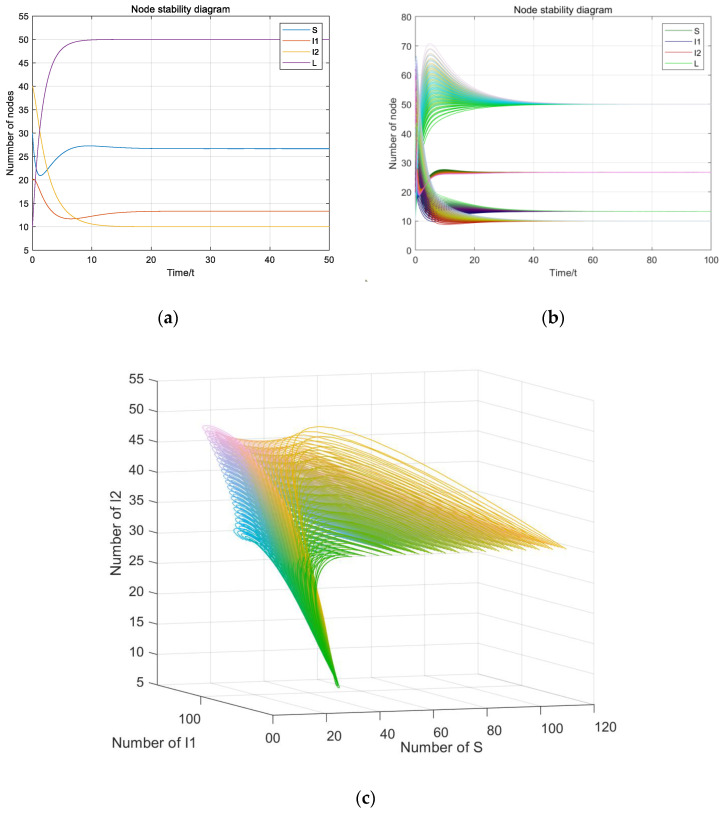
Diagram of the endemic equilibrium: (**a**) Stable value of endemic equilibrium; (**b**) the stability of changes to the initial value; (**c**) three-dimensional diagram of stability of changes to the initial value.

If the parameters satisfy$\;R_{1}>1$ and$\;R_{1}>R_{2}$, $\beta_{1}=0.03,\beta_{2}=0.02$,$\;\gamma_{1}=0.2,\;\gamma_{2}=0.4,c=0.3,u=0.2,\;d=0.1,\;\varepsilon =0.2,\;A=10.$ With the endemic equilibrium $E^{*}\left(S^{*},I_{1}^{*},I_{2}^{*},L^{*}\right)$, we can obtain $S^{*}=$26.7, $I_{1}^{*}=13.3$, $I_{2}^{*},=10$, $L^{*}=50$. The simulation diagram is shown in Figure 6.

Obviously, as shown in Figure 6a–c, it is can be seen that $\;R_{1}=1.125>1$ and $R_{1}>R_{2}=0.75$, and the initial values do not have any influence on system stability and the system (1) will be stable at the endemic equilibrium eventually.

Briefly, the relationship between the system and parameters is shown as a simulation diagram. The rate of ε has a great influence on the stable solution of the system, and other parameters have a great influence on the basic reproduction number to affect the equilibrium solution. Thus, a good system can be constructed by setting reasonable parameters.

### 5.2. Optimal Strategy Simulation

As for the optimal control strategy, the control variable set U($\gamma_{1},\gamma_{2},u)$ is calculated by Pontryagin’s maximum principle, and the numerical result is calculated by implementing a fourth order Runge–Kutta method [33]. Firstly, for the system (1), the nodes’ number of each state can be obtained by initializing control variable values, the time interval is [0,tf] and the transversality condition Equation (35) is satisfied. It is noted that the numerical result of adjoint variables can be calculated by the system (34). Then, by updating U($\gamma_{1},\gamma_{2},u)$ iteratively, the control cost converges to a limited value.

**Figure 7:**
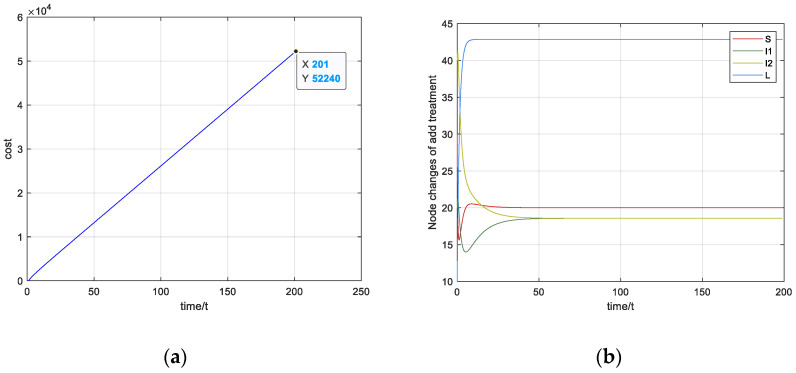
Diagram of cost and node equilibrium without optimal control: (**a**) Cost of the nodes without optimal control; (**b**) the state of equilibrium without optimal control.

The parameter values are given as $\beta_{1}=0.04$, $\beta_{2}=0.03$, c=0.3, d=0.1, ε=0.1, $Q_{I_{1}}=1$, $Q_{I_{2}}=2$ and $Q_{L}=1$. The control variable set U($\gamma_{1},\gamma_{2},u)$ is $\gamma_{1}=0.3$,$\;\gamma_{2}=0.3$ and u=0.3. The simulation diagram of the cost value without optimization control is shown in Figure 7a. At the end of time tf=200, the cost J is 52,244, and the diagram of the equilibrium point is shown in Figure 7b.

**Figure 8:**
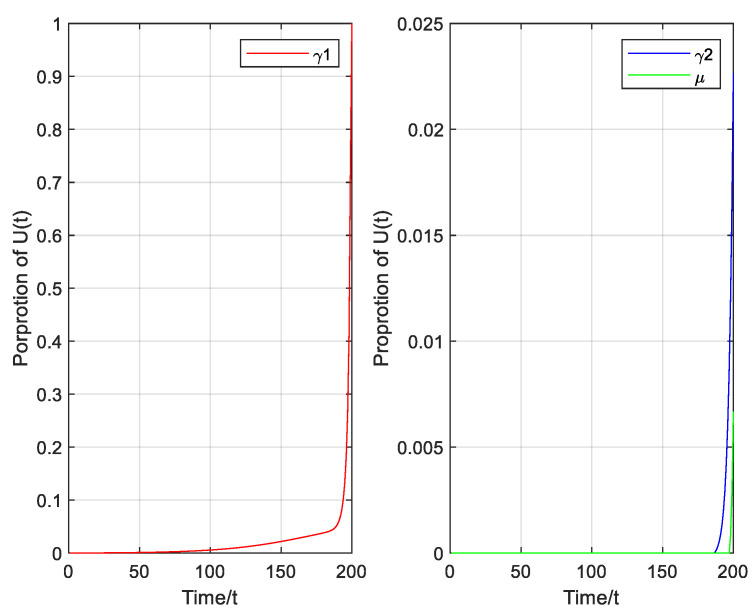
Proportion of control variable set.

As shown in Figure 8 considering the optimal control, the rate of cleaning $I_{1}$ virus (i.e., $\gamma_{1}$) increases to 1 gradually; the rate of cleaning $I_{2}$ virus (i.e., $\gamma_{2}$) is increased to 0.0226 and the rate of recovery energy (i.e., u) increases to 0.0067. 

**Figure 9:**
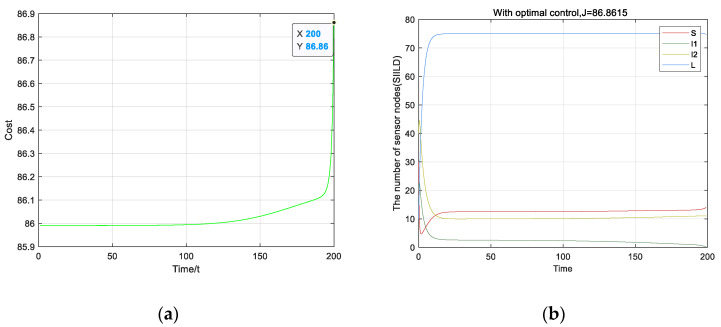
Diagram of the result of optimal control: (**a**) Cost of the nodes of the optimal control; (**b**) the state of equilibrium of the optimal control.

It is noted that the cost under the optimal strategy is greatly reduced, as shown in Figure 9, which is equal to 86.86 at the terminal time tf, shown in Figure 9a. As shown in Figure 9b, the values of $I_{1}$ and $I_{2}$ are smaller than that without optimization control. It is obvious that the performance under the optimal strategy is superior to that without optimization control.

## 6. Conclusions and Future Work

In this paper, a novel epidemic model in WRSNs is proposed, including susceptible, infected, variant and low-energy states. The model considers rechargeable sensors and the virus mutation factor. By analyzing the basic reproductive number, the existence of equilibriums is first proved, and the local stability and global asymptotic stability are proved by the Lyapunov stability criterion. Meanwhile, the influence of the rate of virus mutation and the number of mutated nodes on the endemic equilibrium is revealed. In addition, an optimal strategy is proposed to minimize the numbers of the infected nodes and the virus mutation nodes, the cost of cleaning the virus and the cost of charging low-energy nodes. Finally, the numerical simulation validates the theoretical results.

Future research will attempt to consider the time delay to move closer to practical application. For WRSNs, it is a key topic of future work to consider the time delay of the virus mutation and the time delay of charging at the same time. Additionally, the model can become closer to reality when the time delay is proposed.

## Figures and Tables

**Table 1:** Research on epidemic models which consider virus mutation.

Authors	Research Field	Model	Content
Yang et al. [23]	Dynamic analysis of the virus mutation model	SIS	Proof of the local and global stability of the system
Dong-Mei et al. [24]	Model analysis of disease viruses mutated in the process of transmission	SEIR	Proof of the local and global stability of the system that considers the exposed
Gao et al. [25]	An SEIR epidemic model analysis with logistic death rate of virus mutation	SEIR	Proof of the local and global stability of the system that considers the logistic death rate of virus mutation
Tong et al. [26]	Dynamic model analysis with delay of the virus mutation	SIS	Proof of the local and global stability of the system that considers the time delay
Dong-Mei et al. [27]	SIR model analysis with delay of the virus mutation	SIR	Proof of the local and global stability of the system that considers recovered factor and the time delay
De-gang et al. [28]	A variation epidemic model’s propagation and analysis in complex networks	SIVR	Proof of the local and global stability of the system
Cai et al. [29]	Model analysis of spread of the pathogen with mutant strain and vaccination	SIVR	Proof of the local and global stability and analysis of the Hopf bifurcation of the system
Xu et al. [30]	Optimal control of the SIVRS epidemic spreading model with virus mutation in complex networks	SIVRS	Considers the optimal strategy and calculates the optimal control results of the system

## Data Availability

Not applicable.
